# Dissolved oxygen content prediction in crab culture using a hybrid intelligent method

**DOI:** 10.1038/srep27292

**Published:** 2016-06-08

**Authors:** Huihui Yu, Yingyi Chen, ShahbazGul Hassan, Daoliang Li

**Affiliations:** 1 College of Information and Electrical Engineering, China Agricultural University, Beijing 100083, China; 2 Key Laboratory of Agricultural Information Acquisition Technology, Ministry of Agriculture, Beijing 100083, P.R. China; 3 Beijing Engineering and Technology Research Center for Internet of Things in Agriculture, Beijing, 100083, P.R. China

## Abstract

A precise predictive model is needed to obtain a clear understanding of the changing dissolved oxygen content in outdoor crab ponds, to assess how to reduce risk and to optimize water quality management. The uncertainties in the data from multiple sensors are a significant factor when building a dissolved oxygen content prediction model. To increase prediction accuracy, a new hybrid dissolved oxygen content forecasting model based on the radial basis function neural networks (RBFNN) data fusion method and a least squares support vector machine (LSSVM) with an optimal improved particle swarm optimization(IPSO) is developed. In the modelling process, the RBFNN data fusion method is used to improve information accuracy and provide more trustworthy training samples for the IPSO-LSSVM prediction model. The LSSVM is a powerful tool for achieving nonlinear dissolved oxygen content forecasting. In addition, an improved particle swarm optimization algorithm is developed to determine the optimal parameters for the LSSVM with high accuracy and generalizability. In this study, the comparison of the prediction results of different traditional models validates the effectiveness and accuracy of the proposed hybrid RBFNN-IPSO-LSSVM model for dissolved oxygen content prediction in outdoor crab ponds.

Dissolved oxygen is one of the most important physical properties in crab ponds because it has great influence on the overall health and growth status of the aquatic ecosystem^1^. Proper control and management of dissolved oxygen in crab pond aquaculture is crucial for the developing crabs and has a significant impact on the quality and quantity of the final product^2^.Thus, the efficient and accurate prediction of the dissolved oxygen content in modern aquaculture can provide a basis for water quality control and management, reducing aquaculture risk and financial losses and optimizing operation^3^,^4^. Liu *et al.*^2^ have built dissolved oxygen content prediction models using machine learning methods that use just the complete valid data to build the forecasting model without considering invalid data samples. In a practical application, however, the data provided by a single sensor may lack accuracy or have limits^5^, e.g., missing data or extreme data. Hence, this study presents a new hybrid dissolved oxygen content forecasting model that first uses the data fusion method to improve information accuracy and provide trustworthy training samples and then builds the dissolved oxygen content prediction model.

Irregularities in the data from multiple sensors may be due to incomplete or partial data or human activity^6^.The accuracy of the prediction model relies on the accuracy of the training data. Moreover, the data collected by a single sensor maybe inaccurate or limited^7^. Hence, a good method must be adopted to improve the accuracy of the sensor data. In recent years, different data fusion^5^ strategies such as feature selection, activity recognition, fault detection and precise information provision^7^,^8^,^9^,^10^ have been developed to integrate information from multiple sensors. Data fusion techniques are mathematical techniques used to combine multiple values into a single precise value. The radial basis function neural network (RBFNN) method is one of the artificial neural network methods used for multi-sensor data fusion that has high accuracy^11^. The goal of using data fusion in this research is to obtain more precise data for the dissolved oxygen content prediction model.

In the last few years, different approaches have been applied to water quality prediction. The typical water quality simulation and prediction models can be divided into physical models and black box models. The physical methods are based on mathematical theory^12^,^13^. Thus, it is a difficult task to determine the parameters or extrapolate from the sub-models^2^. In contrast to physical models, ‘black box’ prediction models do not rely on situation-specific details and do not need to determine many parameters. Therefore, there have been many attempts made to use artificial intelligence techniques, such as time series methods, artificial neural network methods, and support vector machine methods^1^,^14^,^15^,^16^,^17^.

Time series methods have been used for linear water quality prediction models, for example, the auto regressive moving average (ARMA) model and the auto regressive integrated moving average (ARIMA) model^18^,^19^,^20^. However, the dissolved oxygen content in crab aquaculture is complicated, changes nonlinearly and is influenced by many factors, hence, some time series methods cannot provide precise prediction accuracy^21^. Artificial neural networks (ANN) comprise a general purpose model that has been used to develop forecasting models with nonlinear series^22^. Hatzikos *et al.* develop neural network models to predict seawater quality indicators such as water pH, temperature and dissolved oxygen content^23^. Ma *et al.* use a back propagation neural network (BP-NN) model to predict water quality for aquaculture water management^24^. Although the neural network models have lower mean absolute error in their predictions and react more evenly to the indicators than their logistic counterparts^25^, ANN also suffers from drawbacks, such as no exact rule for setting the neural network parameters and the time complexity of the learning process^21^. To overcome these disadvantages, a new approach should be explored.

A least squares support vector machine (LSSVM) is a robust regression technique used to solve with few samples and perform nonlinear function regression^26^, so it has been applied in prediction methods for various fields^25^,^27^,^28^. In this study, a least squares version of SVM (LSSVM) is considered, in which the training is expressed in terms of solving a set of linear equations in the dual space instead of quadratic equations, as for the standard SVM case^29^. Moreover, the least squares support vector machine (LSSVM) improves on SVM by applying linear least squares criteria to the loss function^4^,^30^. In addition, the kernel parameter 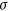 and the regularization parameter 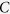 in the LSSVM training procedure significantly influence forecasting accuracy^31^. To achieve a high level of performance with LSSVM models, the key parameters have to be tuned. To date, an exact method of obtaining an optimal set of LSSVM hyper parameters has not been determined.

Particle swarm optimization (PSO) is a heuristic global optimization method introduced by Kennedy and Eberhart in 1995^32^. It is widely used in fields such as function optimization, parameter training, model classification due to its many advantages, including its simplicity and easy implementation^33^,^34^,^35^. However, basic particle swarm optimization does not ensure convergence to an optimal solution and is also prone to partial optimization, which reduces precision in the regulation of its speed and direction^36^. Due to comparatively poor efficiency, a number of studies have been conducted on improving the performance of PSO algorithms, which are used in parameter optimization^2^,^37^. This study presents an improved particle swarm optimization algorithm for simultaneously optimizing the LSSVM parameters.

## Results and Discussion

### Dissolved oxygen content fusion result analysis

All computation for the prediction model was performed in MATLAB by coding in M files. In the proposed algorithm, all the experimental data were obtained from the same crab pond. The meteorological data were collected by the weather station installed on the shore of the pond, and the water quality data were collected from sensors installed in the same pond. All these experimental data were simultaneously transferred to the Digital Wireless Monitoring System of Aquaculture Water Quality and used by the proposed algorithm. The experimental data include water temperature, solar radiation, wind speed, rainfall, humidity, and four dissolved oxygen content sensor readings, which were used for data fusion. Because the data obtained from a single dissolved oxygen sensor is more unreliable, dissolved oxygen content values from dissolved oxygen sensors in four locations were used for data fusion along with other factors to obtain a relatively accurate dissolved oxygen content value. Then, the fused dissolved oxygen content value was used with the water temperature, solar radiation, wind speed, rainfall, and humidity data as the input sample for machine learning.

In the fusion method, we applied the RBF algorithm to the four DO sensors’ data to obtain a better forecast-model training sample. The dissolved oxygen content value obtained by sensor1 was accepted as the real dissolved oxygen content value. For this part of the study, we used the first 500 data values from each of the four DO sensors as the fusion training samples and the next 200 as the test samples. Then, the water temperature, solar radiation, wind speed, rainfall, air humidity, and dissolved oxygen content values (from the 200 test sample groups) used for fusion were used as the prediction training and test samples for the dissolved oxygen content forecasting model.

**Figure 1:**
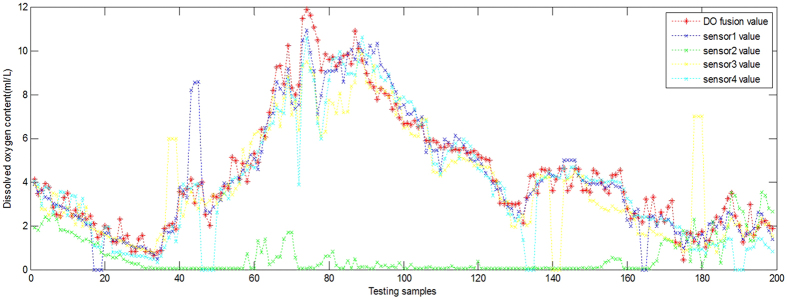
Fusion data and the original sensors’ dissolved oxygen content value.

After development, the RBFNN method was used to fuse or integrate the data; Fig. 1 shows the result of the data fusion. According to Fig. 1, there are many invalid (zero) and distorted (much higher than those nearby) dissolved oxygen content values in the original data. There is even one dissolved oxygen sensor (sensor 4) reporting data that is too low. However, fusion on the dissolved oxygen content data can effectively eliminate low credibility data. The data plots show that the fusion data discounts the bad sample data and generates more credible samples (Fig. 1).

Table 1 shows several invalid and distorted data values among the original dissolved oxygen content values obtained by the four DO sensors. As shown by the table, the results indicate that the fusion method can train with the other input factors to fuse data to obtain more trustworthy data for the prediction model. For example, at 20:20, the dissolved oxygen content value from sensor 1 is valid, and at 10:20, the dissolved oxygen content values from both sensor 2 and sensor 3 are invalid. The fusion values are more accurate than the invalid values. As the accuracy of the prediction model is dependent on the training samples, the fusion method is suitable to obtain more reliable data.

### Forecast results analysis with fusion data

In the dissolved oxygen content forecasting model, the water temperature, solar radiation, wind speed, rainfall, air humidity, and previous fusion dissolved oxygen content value (test sample data) were combined for use as the prediction training and test values. The first 120 groups of data were used as training data and the last 80 groups of data were used as test data. To compare and evaluate the regression results of the RBFNN-IPSO-LSSVM dissolved oxygen content prediction model, we also used the BP neural network and standard LSSVM methods to predict the dissolved oxygen content. To analyse and compare prediction performance, the BP neural network, standard LSSVM and the optimized LSSVM all used the fusion data as training samples and forecast the sequence fusion test samples for the same time period. The BP neural network method consists of six input variables and one output variable, a hidden layer with five initial neurons, and a maximum training step value of 10^4^. The standard LSSVM model parameters were selected by a 5-fold cross-validation method.

**Figure 2:**
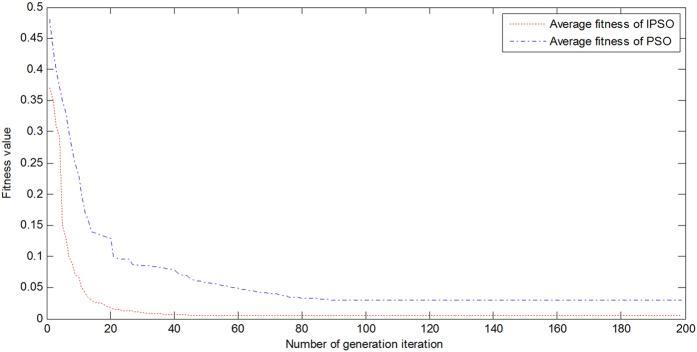
Fitness performance for the proposed IPSO and the traditional PSO.

In the optimized LSSVM, the parameters were optimized by the improved PSO algorithm. The population size of the traditional PSO and the improved PSO were set to 50, the maximum evolution generation was set to 200, the particle dimension is 2, the initial inertia weight 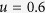, and in the improved PSO the mutation probability 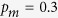 and 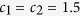. The fitness performance graph in Fig. 2 shows the fitness curves decrease rapidly at the outset of a generation but soon level off. The optimal parameters selected for the LSSVM by the improved IPSO were 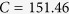, 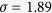.

**Figure 3:**
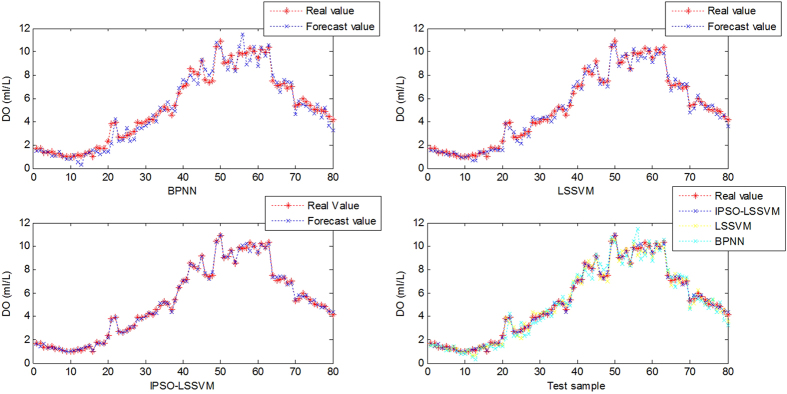
The dissolved oxygen content forecasting value of the RBFNN-IPSO-LSSVM in contrast with the comparison models.

In this step, the fused dissolved oxygen content data were used as the real data for the three comparison methods’ samples. Figure 3 contains the forecasting results of the BP, LSSVM and IPSO-LSSVM methods. The results show that the proposed hybrid model is more suitable and effective for dissolved oxygen content prediction. It has a strong ability to learn using a small nonlinear sample to achieve excellent generalizability.

As different algorithms employ different experimental methodologies, this study uses different standard statistical performance evaluation criteria. The standard statistical criteria include the root mean square error (RMSE), the mean absolute percentage error (MAPE), the Nash-Sutcliffe efficiency coefficient (NSC), the coefficient of determination (R^2^) and the running time (T). Table 2 shows the error index for the three models. It indicates that the IPSO-LSSVM model combining the improved particle swarm optimization algorithm with the least squares support vector machine hybrid model is more adequate than the standard LSSVM and the BP neural network. The index (MAE, RMSE, MSE, NSC and T) of the IPSO-LSSVM is better than those of the other models. The running time (T) of the IPSO-LSSVM model is less than the LSSVM model, indicating the improved PSO method effectively selects the parameters of the LSSVM. Using the same test data, the relative MAE, RMSE and MSE differences between the IPSO-LSSVM model and the LSSVM model were 34.63%, 47.62% and 60.14% in the testing period. The relative MAE, RMSE and MSE differences between the IPSO-LSSVM model and the BP neural network model were 51.28%, 54.69%, and 67.32% in the testing period. So the IPSO-LSSVM model is more able to solve the solar greenhouse temperature prediction problem than the SVM and BP neural networks. Moreover, the NSC of the IPSO-LSSVM is higher than that of the other models. It is obvious that the IPSO-LSSVM model has significantly more reliable performance and generalizability and a higher prediction accuracy than other models.

## Conclusions

In this study, a novel hybrid algorithm has been proposed for dissolved oxygen content prediction in outdoor crab ponds. To remove redundant and erroneous data from the original data, the RBF neural network method is used to fuse the original data to prepare training samples for the prediction model. Then, improved particle swarm optimization is used for the selection of the parameters for least squares support vector regression. Obtained results show that the proposed model yields better prediction accuracy in comparison with several other machine learning methods. Looking at the fusion result, we can see the fusion method removes erroneous data, increasing the original data’s reliability, leading to a more trustworthy training sample for the prediction learning machine. The forecasting results show the IPSO-LSSVM model predicts more accurately than the traditional models. The proposed hybrid model is used to predict the dissolved oxygen content in outdoor crab ponds. Our results demonstrate that the RBFNN-IPSO-LSSVM prediction model is effective and feasible.

For further study, the influence of some factors such as water temperature, wind speed, and solar radiation on the dissolved oxygen content is unclear. Hence, a method that can determine which factors should be used as input is important for improving the accuracy of the prediction model. Additionally, to obtain more precise hybrid predictor methods, the combination of different signal processing tools, feature selection and learning machines may be examined.

## Materials and Methods

### Data preparation

**Figure 4:**
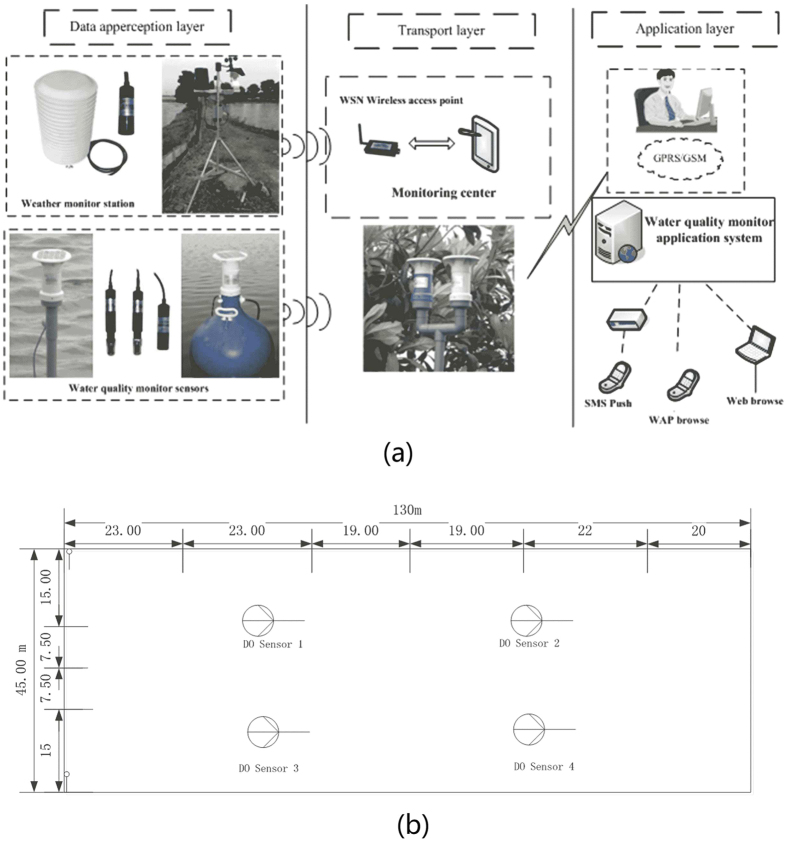
Experimental data collection system and location: (**a**)The structure diagram of the digital wireless monitoring system; (**b**) The sensor layout in the river crab pond. The photographs of the man, computer systems and web browser in Fig. 4 were taken by first author Huihui Yu in Gaocheng town, Yixing city, Jiangsu province. The drawing of the man in the top right of the figure, and the drawing of the equipment next to the “Water quality monitor application system” were created by Yingyi Chen. And, the whole figure was designed and drawn by author Huihui Yu and Yingyi Chen.

The data used in this study were collected by the Digital Wireless Monitoring System of Aquaculture Water Quality. Figure 4(a) shows the system structure diagram. The system is applied in more than 1000 river crab farming ponds, approximately 10000 acres. The system is made up of three major parts: the data acquisition layer; the information transport layer; and the application layer. The data acquisition layer is comprised mainly of the water quality monitoring sensors, such as the pH sensor, DO sensor, salinity sensor, and the weather monitoring station for temperature, solar radiation, atmospheric humidity, and wind speed. All of the data are moved via the transport layer to the application layer for data apperception, intelligent information processing, and logical operations.

In this study, the water quality and meteorological data were obtained from 21 June to 12 July in intervals of twenty minutes, totalling more than 1000 samples. In this study, the experimental details are as follows: (a) The size and population density of the crabs. During late June and early July, crabs complete their third shelling and begin the growth process for the fourth shelling. During this period, the stocking density of crabs is approximately 2000 per acre, their average weight is approximately 75 grams, and they reach a diameter of approximately 5.0 cm. (b) The area of the pond. The crab pond length is 130 meters and the width is 45 meters, for a total area of 5850 square meters. (c) The location of the sampling sites. The dissolved oxygen content collection sites are all in the same crab pond. The four dissolved oxygen sensors are evenly distributed in the crab pond; the specific locations are shown in Fig. 4(b). The meteorological data are collected by the weather station installed on the shore of the pond. These collection data include water temperature, solar radiation, wind speed, rainfall, humidity, and the dissolved oxygen content values from the four dissolved oxygen sensors. All these experimental data are simultaneously transferred to the Digital Wireless Monitoring System of Aquaculture Water Quality and used for the proposed algorithm.

### The structure of the prediction model

**Figure 5:**
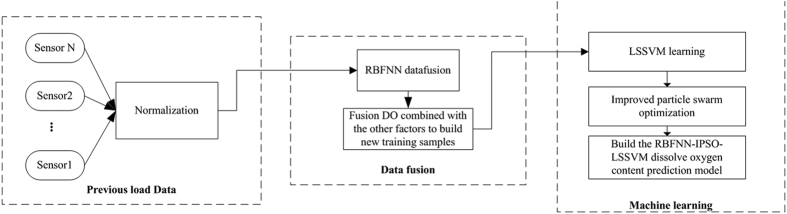
Proposed algorithm for dissolved oxygen content forecasting.

Before training the machine learning model, we need to pre-process the original dissolved oxygen content data using the RBF neural network. Then, the improved particle swarm algorithm is used to determine the kernel parameter 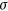 and the regularization parameter 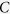 for the least squares support vector machine. Finally, the dissolved oxygen content prediction model is built by training the forecasting method. As depicted in Fig. 5, the proposed method consists of three main parts:

- Data fusion.
- Least squares support vector regression parameters selection.
- Training of the learning machine.

### Multi-sensor Data fusion by RBF neural network

The radial basis function (RBF) neural network is one of the neural network models that learn by measuring Euclidean distance data^38^. The RBF neural network model has a three-layer structure: the input layer, the hidden layer, and the output layer^39^. Movement from input layer to hidden layer is nonlinear, and that from hidden layer to output layer is linear. Determining the number of hidden nodes is an important issue that has a substantial impact on the neural model quality^40^. The input 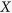 is an M-dimensional vector, 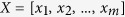. The input layer units are only distributed to the hidden layer^41^.

In the RBFNN method, each neuron in the hidden layer has a Gaussian function described as:


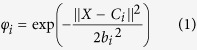


where 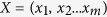 is the 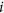 th input element, 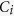 is th centre of the 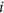 th hidden unit, 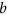 is the width of the receiving field, and 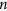 is the number of input elements.

**Figure 6:**
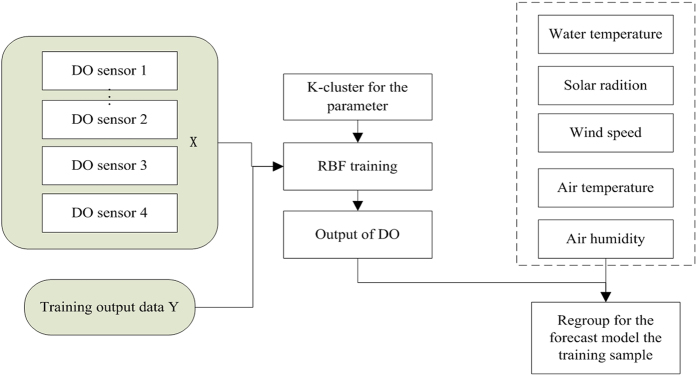
Process of multi-sensor data fusion by the K-cluster RBF method.

The output layer is activated by the linear combination of the hidden layer units, which can expressed as:


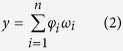


where 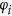 is the weight of the connection from the hidden layer to the output layer. The RBF neural networks adopt the K-means clustering algorithm to improve the fusion result. The RBF neural network process for multi-sensor data fusion is shown in Fig. 6 and can be described as follows:

Step 1: Select the input vectors 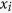 as the training sample, 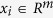 represents each group of data coming from all 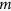 detectors;

Step 2: Train the RBF neural networks by the K-means clustering algorithm and select the parameters;

Step 3: Set the error value and run the algorithm until the termination criterion is satisfied;

Step 4: Get the fused dissolved oxygen content value from the RBF neural network, then combine the environmental data and the fused dissolved oxygen content value into a new sample for the IPSO-LSSVM.

### Least squares support vector machine (LSSVM)

The least squares support vector machine (LSSVM) has been introduced as a reformulation of the standard support vector machine (SVM), which has simple techniques^42^,^43^. The LSSVM can address linear and nonlinear systems with structured risk minimization, and it has been successfully applied in many fields. In an LSSVM model, the training dataset is assumed to be 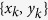

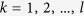
, where 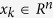 is an input vector and 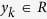 is its corresponding target vector. The regression problem can be transformed into the following optimization problem:


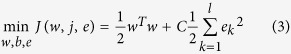



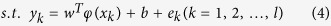


where 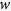 is the weight vector, 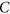 is the regularization parameter, 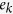 is the error between the predicted and actual values of the system and 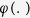: 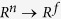 is a function used to map the input space to a higher dimensional space. Using the Lagrangian function, the LSSVM model is written as follows:


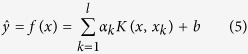


where 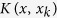 is the kernel function. In this study, the RBF was selected as the kernel function because the RBF ably handles nonlinear relationships and its overall performance is excellent. This method maps the sample to a high dimensional space in a nonlinear fashion and it has few required parameters; therefore, it is the most popular option for kernel function. The kernel function is shown in Eq. (6):


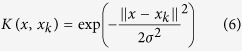


The model can be written as follows:


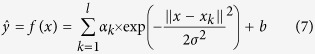


### Improved particle swarm optimization

The algorithm for particle swarm optimization (PSO) is an evolutionary optimization algorithm. It is initialized with a group of 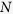
random particles and simulates social behaviour among individuals^44^,^45^. The position of particle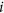 is represented as 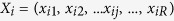. The velocity of particle 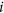 is represented as 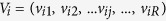, where 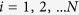 and 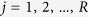. The best previous position of particle 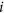 is represented as 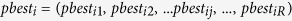 and the best particle among all particles is represented as 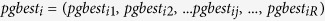. 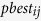 is the local best position of the particle 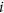 in the 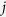 th dimension, and the 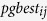 is the global best position of the swarm in the 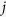 th dimension. During the search process, the direction of each particle is adjusted by dynamically altering its velocity according to both its own movement and that of neighbouring particles^46^,^47^. The particle position and velocity are updated according to the following equation:


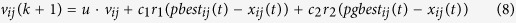



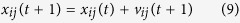


where 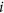 is the iteration counter, 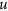 is the inertia weight, which is used to control the impact of the previous history of the current particle, 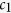 is the cognition learning factor, 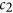 denotes the social learning factor, and 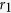 and 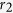 are uniformly distributed random variables within the range [0,1].

The original particle swarm optimization has a slow convergence and may only find the traditional local optimum. In the study, an improved particle swarm optimization is presented to find a suitable inertia weight to balance the local and global search abilities. The dynamic adjustment method of the inertia weight 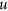 is written as Eq. (10):


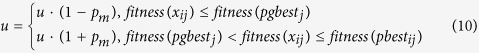


The 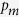 is introduced in this study as a mutation probability used to change the inertia weight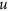. When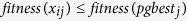, the particles have a status which is close to the global optimum, so they are given a smaller inertia weight than the current one. The smaller inertia weight can help the particles to reach optimum status more quickly. When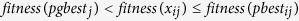, the particles are not close to the global optimum, and therefore, a bigger inertia weight is needed to change their position and velocity. The improved particle swarm optimization increases the convergence rate and also improves the accuracy of the solution.

### Parameter selection

For least squares support vector regression, the kernel parameter 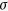 and the regularization parameter 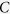 in the LSSVM training procedure have significant influence on forecasting accuracy. The improved particle swarm optimization is devoted to optimizing the kernel parameter 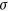 and the regularization parameter 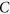. Each particle represents a potential solution of the vector 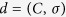. The fitness function represents the performance of each particle and the fitness function is defined in the model as follows:


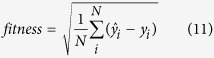


where the 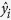 represent the predicted values, the 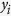 represent the actual values, and 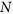 represents the size of the predicted value subset. The particle with a minimal fitness value is the global extreme point. The process of optimizing the LSSVM parameters with IPSO can be described as follows:

Step 1: Particle initialization and IPSO parameter setting: generate a population of initial particles that consists of parameter 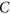 and kernel parameter 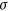. Set the maximum number of iterations 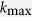, the particle population number 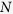, and the minimum fitness value for error limitation.

Step 2: Set the iteration variable 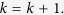


Step 3: Calculate the fitness function value of each particle using Eq. (11): use the current particle as the individual extreme point of every particle and the particle with the minimal fitness value as the global extreme point.

Step 4: Calculate the weight 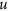
using Eq. (9), then update the velocity and position of the particles according to Eqs (8)–(9), 55.

Step 5: Stop the algorithm if the termination criterion is satisfied and the best LSSVM model is produced. Otherwise, return to Step 2.

## Additional Information

**How to cite this article**: Yu, H. *et al.* Dissolved oxygen content prediction in crab culture using a hybrid intelligent method. *Sci. Rep.* **6**, 27292; doi: 10.1038/srep27292 (2016).

## Figures and Tables

**Table 1:** Error data fusion results obtained with the four DO sensor value-based RBF neural network data fusion methods.

Time	Dissolved oxygen content				
	Sensor 1	Sensor 2	Sensor 3	Sensor 4	Fusion value
29-06-2015 20:20	0.00	1.21	1.72	1.05	1.4547
30-06-2015 02:40	1.72	0.06	6.00	1.97	2.0888
30-06-2015 03:00	2.34	0.06	6.00	1.28	1.8274
30-06-2015 14:00	7.43	0.06	6.38	3.87	8.4344
01-07-2015 09:20	3.20	0.06	2.03	0.00	3.2813
01-07-2015 10:20	4.28	0.06	0.00	4.25	4.1231
01-07-2015 18:40	0.00	0.06	1.64	2.40	2.1536
02-07-2015 03:00	1.40	1.40	1.51	0.00	1.2531
02-07-2015 05:40	1.16	1.16	7.00	0.89	1.6136

**Table 2:** Error statistics of four forecasting models.

Model	MAE	RMSE	MSE	NSC	T
IPSO-LSSVM	0.2814	0.4057	0.1085	0.9531	3.2143
LSSVM	0.4305	0.7745	0.2722	0.9187	3.1265
BPNN	0.5776	0.8954	0.3320	0.9002	4.3298
